# Philosophical and distinct SLE epitomes: dogmas in conflict with evidences and an intellectual dissonance between established pathophysiological models

**DOI:** 10.3389/fimmu.2025.1580664

**Published:** 2025-07-24

**Authors:** Ole Petter Rekvig

**Affiliations:** ^1^ Section for autoimmunity, Fürst Medical Laboratory, Oslo, Norway; ^2^ Department of Medical Biology, Faculty of Health Sciences, UiT The Arctic University of Norway, Tromsø, Norway

**Keywords:** dogma definition, dogma identification, evidence-based facts, systemic lupus erythematosus, lupus nephritis, DNA structure diversity, chromatin autoimmunity

## Abstract

This study centers around dogmas, their identifications and definitions, and their impact on our understanding of what Systemic lupus erythematosus (SLE) is. A focus is centered on description of how we investigate this enigmatic syndrome, and how we try to describe processual elements that can be targeted by experimental therapy modalities. Mostly, this study deals with definitions and critical insight into how dogmas hinder our understanding of SLE. When we start to investigate apparently convincing statements related to SLE, it is surprising how many of them are uncovered as authoritative, but not founded by concrete evidence! This problem refers to a definition of a dogma: *A point of view or tenet put forth as authoritative without adequate grounds or evidence (Merriam-Webster).* For example, several central statements/criteria are revealed as dogmas that challenge our insight into SLE as a complex syndrome. Critical in this context is the immense impact of “SLE classification criteria” versions in relation to evidence-based basic SLE processes. The SLE classification criteria will, as described in this study, most probably not identify SLE as “a one disease entity,” but more likely as a “poly-causal, poly-etiological, and poly-phenotypic “theoretical template SLE,” “SLE-like,” or “SLE-like non-SLE” syndromes. This is problematic as SLE may, in context of definitions described here, not be rationally structured by classification criteria. This prevents SLE cohorts from being suitable and ideal as study objects aimed to investigate experimental therapy modalities, genetics, etiology, and pathophysiology. However, this pessimistic view may turn into optimism if dogmas described in this study are identified and subjected to causal studies based on critical hypotheses. Today’s interpretative use of SLE classification criteria tentatively maintains a narrative that describes scientific studies of the SLE syndrome as not optimal and not ideal.

## Introduction

“The SLE Classification criteria” are formulated and proclaimed to categorize SLE as a disease entity (1, 2). From arguments discussed below, this proclamation is logically rather deficient and promotes an easy to understand paradox: The SLE classification criteria declare on one side that they define SLE cohorts as useful study objects; on the other hand, however, SLE classification criteria promote in practice SLE as “an enigmatic, prototypic autoimmune syndrome” [discussed in (3, 4)].

Think it over and consider if it is true: The classification criteria do not define SLE as one complex disease entity but open also for a group of poly-phenotypic and poly-causal (SLE-like non-SLE) syndromes (5, 6). How should we then logically handle this paradoxical problem, and how shall we redefine, handle, and implement new critical and realistic research hypotheses?

In order to study complex problems as implicated in the enigmatic syndrome SLE, we have to identify what we know about SLE based on scientific evidence. Even more important, it is important to identify what we theorize based on statements and hypothetical proclamations suggested by “the relevant scientific establishments.” This points at a theoretical conflict between *(i.)* evidence and *(ii.)* assumptions and theoretical principles. The latter represents authoritative trend-setting dogmas bringing presumptions to the discussion/debate forum.

*Authoritative presumptions principally inherit inconsistent intellectual qualities as long as presumptions substitute for factual evidence-based knowledge.* This reasoning is very close to a definition of a dogma, and, thereby, the dogma may conquer the scientific landscape by indicative assumptions rather than evidence-based scientific solidity! Missing evidence renders dogmas scientifically counterproductive, practically defining an inherent scientific problem in SLE research. This reasoning inherits imperative hypotheses requiring identification of “dogmatic spots” in the scientific SLE landscape. For example, SLE classification criteria, diagnostic impact of anti-dsDNA antibodies, and causal interactivity of classification criteria in sense of causality cascades (see below) are all examples of authoritative dogmas based on low levels of evidence, here synonymous with indications. *If we interpret that a dogma expresses something real and correct, then we accept the hidden unofficial although authoritative arguments. They cannot, however, envisage a truth as long as evidence for its reality is missing!* This leads us into an often unexpressed theme in this philosophically intuitive problem: the imperative self-criticism when we are in the border landscape between authoritative dogmas and evidence-based insight. We try to understand theories authoritatively expressed by dogmas, rather than modestly accepting alternative interpretations and open-minded critical research hypotheses.

If we mistrust an authoritative dogma, then we have often no firm explanation for its alternative—we cannot construct a mirror image of an abstracted, non-evident complex theoretical model. *We remain ignorant if we monotonously support a dogma—or uncritically discard a dogma—without thorough logic analyses and discussions.* We need to consider lack of evidence that will and must reduce the dogma’s impact. A dogma may thus leave us with questions, mistrust, and confusing information. *This means in science that we have to precisely identify dogmas, ask critical questions, sort out misconceptions, and put the dogma into a scientific context—into its role as object for critical, unemotional, and courageous research programs. We have to identify dogmas and to pursue them in strict critically scientific contexts.* This is relevant for SLE as a disease characterized by dogmatic explanations and transformations, enigmas, and skepticism.

*A concrete and scientifically evidence-based example: Over decades, a dogma pronounced that mammalian dsDNA was non-immunogenic; later, a new dogma took over; dsDNA was immunogenic, and anti-dsDNA antibodies became an autoantibody population that was an essential and unique diagnostic marker for SLE; subsequently, this dogma transformed into the (yet final) status as an autoimmune product relevant in SLE, infections, and malignancies [thoroughly discussed in* (7, 8)].

The following text is based on a scientific analysis of these contradictory paradigms: How do we understand SLE and how do we define this syndrome when diagnosed by unproven dogmatic proclamations and circumstantial indications—and not at least, how do we accept that the “cause-effect” paradigm in selection of SLE classification criteria has been ignored and not considered essential? This is systematically discussed below.

This study attempts to define the responsibility that we have to identify dogmas and to contemplate the role of dogmas and their instinct for assumption in conflict with evidence or critical hypotheses (9–11).

## Research is influenced and distracted by dogmas

A definition of a dogma may be formulated as a settled idea with authoritative impact and may even define interpretative rules to solve a complex problem (11, 12), although evidence for this potentially important position is missing (13). This definition identifies a dogma to serve as a distraction phenomenon that may be harmful if intervening with scientific studies of complex problems or syndromes—as SLE. Evident in this context, it is important to identify a problem, to study it, and to delimit it by productive and critical science-based hypotheses.

### Definition and logic problematization of the term dogma

A dogma pertains to something in between a postulation or hypothesis and an evidence-based reality. A dogma is defined as a statement put forth as authoritative, without adequate explanations or evidence-based justifications. *Dogmas consistently demonstrate an unrelenting and inherent resistance to be reformed, reinterpreted, or, ultimately, abandoned.* It may paradoxically be more difficult to turn down a dogma than to prove its correctness [ (9, 14); see, e.g., a discussion on anti-dsDNA antibodies in (7)], because a dogma may simply proclaim an unproven statement, whereas evidence for the truth’s reality may be difficult to develop. There are many examples that confirm this theorem.

Dogmas have two principally different derivations. For the first (and most relevant for this study), a dogma may arise from an immanent and concrete physical problem with an apparently sound and logic explanation, however, with lack of its strong argumentation or stringent evidence. Secondly, a dogma may express something linked to an abstracted (religious/sacral) belief that principally cannot be materialized and understood by evidence. The latter alternative will not be further problematized or discussed in this study.

A dogma (as described in the first alternative above) may, in general, arise from a factual and authentic problem that is difficult to understand and to explain. In fact, evidence may both confront and attract researchers differently and transform disagreements into being either counterfactual and illusory or sound and attractive.

### Dogmas versus evidence-based facts—definition of an intellectual conflict with cogent consequences

A paradigm aimed to understand SLE is either based on hypotheses or on critical evidence—in other words, a schism between dogmas and facts.

In a philosophical context, the *distinction between dogmas and facts* is a clear expression of what we concisely need to identify—we have to scientifically approach dogmas (which are hypothetical) and facts (which are concrete and evidence-based) differently.

Selection of SLE classification criteria is based on tentative insight and general knowledge as described in the relevant literature [see, e.g. (2, 5, 15–22),]. Relevance of present and former SLE classification criteria versions is statistically tested by comparing classification criteria against a “template” (as a theoretical, or archetypical, prototype) SLE and against former SLE classification criteria versions developed by similar intellectual procedures (Delphi panels). These analyses have demonstrated a significant reiteration of classification criteria if we compare the four dominant SLE classification criteria versions: the 1971 preliminary SLE classification criteria, the 1982 ACR, the 2012 SLICC, and the 2019 EULAR/ACR SLE classification criteria versions [discussed in details in (13, 23); see below and data in **Table 1**].

**Table 1:** Comparison* of SLE classification criteria in four different classification versions from 1971–2019**.

1971 preliminary SLE Classification criteria	1982 ACR SLE Classification Criteria	2012 SLICC SLE Classification criteria	2019 EULAR/ACR SLE Classification criteria^#^
1 .Facial erythema (butterfly rash) 2. Discoid lupus erythematosus 3. Raynaud phenomenon 4. Alopecia 5. Photosensitivity 6. Oral or nasopharyngeal ulceration 7. Arthritis without deformity 8. Lupus erythematosus cells 9. Chronic false-positive serologic test for syphilis 10. Profuse proteinuria 11. Cellular casts 12. Pleuritis or pericarditis 13. Psychosis or convulsions 14. Hemolytic anemia or leukopenia or thrombocytopenia	1. Malar rash 2. Discoid rash 3. Photosensitivity 4. Oral ulcers 5. Synovitis 6. Serositis 7. Neurologic manifestations 8. Renal manifestations 9. Hematologic manifestations 10. Immunologic manifestations: anti-DNA/anti-Sm antibodies; anti-phospholipid antibodies* 11. ANA	**Clinical Criteria:** 1. Acute cutaneous lupus 2. Chronic cutaneous lupus 3. Oral ulcers: palate 4.Non-scarring alopecia 5. Synovitis involving two or more joints or tenderness in two or more joints 6. Serositis 7. Renal disorder 8. Neurologic disorder 9. Hemolytic anemia 10. Leukopenia (< 4,000/mm3 at least once) 11. Thrombocytopenia (<100,000/mm3) at least once **Immunological Criteria:** 1. ANA above laboratory reference range 2. Anti-dsDNA above laboratory reference range 3. Anti-Sm 4. Antiphospholipid antibodies* 5. Low complement 6. Direct Coombs test	**Obligatory entry criterion antinuclear antibodies** 1. Constitutional fever 2. Acute cutaneous lupus 3. Subacute cutaneous or discoid lupus 4. Oral ulcers 5. Non-scarring alopecia 6. Joint involvement 7. Pleural or pericardial effusion 8. Acute pericarditis 9. Proteinuria > 0.5 g/24 h 10. Renal biopsy class II or V lupus nephritis 11. Renal biopsy class III or IV lupus nephritis 12 .Delirium 13. Seizure 14. Psychosis/delirium 15 .Autoimmune hemolysis 16 .Leukopenia 17. Thrombocyopenia 18. Anti-dsDNA antibodies 19. Anti-Sm antibodies 20. Anti-cardiolipin or anti-ß2GPI or Lupus anticoagulant 21. Low C3 or low C4; Low C3 and Low C4

*This table demonstrates a comparison between the four major SLE classification criteria that appeared from 1971 till 2019. In this table, only criteria without comments or weighted values are given.

**Color code:

• Criteria written in brown, Raynaud phenomena, are present only in the1971 preliminary SLE classification criteria.

• Criteria written in green are unique for the 1971 Preliminary SLE classification criteria, the 2012 SLICC, and the 2019 EULA/ACR SLE classification criteria.

• Criteria written in blue are unique for autoimmunity and inflammation and indicated in the 1982, 2012, and 2019 SLE classification criteria.

• Criteria written in red are shared by the 1971 and 1982 criteria sets.

• Those criteria written in black are shared by all four criteria sets. Criteria may here be designated differently although they express the same. For example, “Renal manifestations,” criterion # 8 in the 1982 ACR criteria, is in the 2012 SLICC criteria designated as Renal disorder (criterion # 7) and in the EULAR/ACR criteria denoted as Proteinuria > 0.5g/24h (criterion # 9), Renal biopsy class II OR V lupus nephritis (Criterion # 10), and Renal biopsy class III OR IV lupus nephritis (Criterion # 11). These versions of criteria contain many of the same individual classification criteria and are differently annotated. These differences reflect increased insight into each criterion and thereby different annotations, and they express the same contextual meaning.

^#^In the EULA/ACR SLE classification criteria presented in this table, only individual criteria are given. For domains, see (18).

## Central SLE-related dogmas that confuse basic and clinical investigations of SLE—the inconsistent SLE classification criteria

SLE is a syndrome that is not easy to understand, not easy to delimitate, and, therefore, not easy to settle as an exact diagnosis (4, 5, 20–22, 24). Because of the difficult-to-describe nature of SLE, it has been, and is still, relevant to designate the syndrome with the following non-stringent idiom: “SLE is a prototype autoimmune, enigmatic syndrome.” Although subjected to intense scientific investigations, this idiom preserves the unresolved enigmatic status of the syndrome. Important in the context, SLE may be defined as enigmatic because the syndrome is characterized by dogmas and eclectic evidence-based facts. If we aim to obtain a better understanding of SLE, dogmas must be identified, delimitated, and subjected to stringent, sound, and foremost critical scientific investigations. *The most central and important dogma that forms our interest in SLE is the “SLE classification criteria.”*

### SLE classification criteria fulfill the definition of a dogma; they are authoritative, but principles underlying their selection are not evidence-based and do not convey the causality principle, and they overrule diagnostic criteria

*A thesis: “Classification criteria have not served SLE research well: There is no evidence in favor of this statement, but there is no evidence in favor of the opposite either; namely, that classification criteria have served, and still serve, SLE research well.”*

This conflicted and paradoxical paraphrase expresses the concept for this study. The main purpose is to distinguish dogmas from their evidential counterparts that are based on science-based insight. Furthermore, it is central to evaluate their alleged origins and to identify conflicts and inconsistencies indicating that dogmas inherit *accepted* authoritative influences in science. This is in opposition to the fact that their impact have not been scientifically and decisively settled [discussed in (5)]. In this context, dogmas may disrupt science-based strategies, leaving us with a great deal of confusions and uncertainties. This provides us with a focus that may enable us to propose and analyze origin and impact of a dogma, evolved and designated as, e.g., “The SLE Classification Criteria.” Other potential dogmas linked to SLE and SLE research will be discussed below in light of the authoritative status of “The SLE Classification criteria.”

We must in the following text comprehend the conception: *We must let the evidence talk, but remember: Lack of evidence has also a message to give!*

All serious science disciplines are critical, intuitive, and reflective and must remain so. We are using a vast amount of energy, intellectualism, and resources to confirm, defend, and explain our theoretical SLE models (6, 19–22, 24–28). More precisely, we aim to determine if current models factually are correct and to describe if they reflect a real link to causality and, consequently, to etiology, pathophysiology, and genetics that, in sum, form the SLE syndrome paradigm [discussed in (6, 13)]. *Do we from these considerations paradoxically aim our efforts to conserve a potentially erroneous SLE-related definition and a concise description*? Do we have strong arguments to implement dogmas as delimiters for definition of well-defined templates, e.g., relevant for standardized and classified SLE? *This conflict may adversely maintain and promote survival of rigorous and enduring SLE-associated dogmas*.

The basis for these problems relies mostly on the principal procedures that account for evolution of the SLE classification criteria. These criteria inherit three serious problems that hamper penetrating insight into the syndrome:

- For the first, the classification criteria overrule and preclude SLE diagnostic criteria. This is true, although concise arguments are not given that explain why classification criteria exclude implementation of diagnostic criteria. From the following argument, this is problematic. Diagnostic criteria are linked to etiology and basically to *causality principle and the causality cascade (see below)*. Classification criteria do not reflect implementation of the causality principle (29, 30) and ignore the causality cascade (13, 31, 32). This is true because these criteria are selected on basis of unfocused SLE-related, but not SLE-identifying, randomly selected parameters. They are selected intuitively on the basis of experience and insight presented by SLE experts belonging to different but relevant scientific disciplines. The causality principle has not been and is still not a focus in SLE classification criteria publications (2, 15–18, 23, 33–35).
  On the other hand, the impact of classification criteria is compromised in absence of basic experimental or clinical observational evidence that, from a philosophical point of view, could indeed also support evolution of SLE diagnostic criteria (see below).
- Secondly, SLE cohorts are established according to the classification criteria attribution rules. These cohorts are preferentially established with the aims to investigate central aspects that, in sum, make up the syndrome SLE, like experimental therapeutic modalities, genetics, etiology, and pathophysiology (15–18). There is one problematic aspect linked to this unsophisticated research plan and experimental protocol(s). The study object, the SLE cohorts, may be poly-phenotypic and poly-causal by nature (13). *Evidence against this view has not been provided.* These facts represent arguments against the idea to analyze aspects of the SLE syndrome using heterogeneous SLE cohorts as study objects.
- For the third, what is the evidence that SLE classification criteria identify SLE as “a (genuine) one disease entity” (2)? Has clear evidence for this demanding paradigm been presented and comprehended? This conflict leaves the critically important SLE classification criteria open for qualified and critical investigations based on an unexpected hypothesis:
- *SLE classification criteria fulfill the definition of a dogma*. The criteria are authoritative by nature, but evidence for their impact related to identification of a delimitated (template) SLE is still missing. Evidence for the opposite view also remains obscure (5, 21, 23). Therefore, the classification criteria may be funded on dogmatisms deriving from unstructured and poorly defined Delphi panel strategies (2, 36, 37).

### Problems that adhere to the procedures providing us with SLE classification criteria—an equation and some critical comments and confrontations

A large panel of experts on various aspects of SLE selected and subsequently elected SLE classification criteria in harmony with standard Delphi panel procedures (36, 37). A fundamental question is relevant to ask: How are underlying protocols defining rules for the selection principle of SLE classification criteria? Most importantly, where can we read the details about these central delimitations?

### The basic and plain SLE criteria selection processes may be described by a philosophical equation in two contrasting interpretative versions

**Version 1 and its interpretation**

*A (symbolizing “criteria”) is statistically associated with B (symbolizing SLE) because B is the factor that promotes A^1^*.

The central problem (and challenge) in this equation is how SLE (B in the equation) will be identified to serve as an authoritative SLE prototype (from now on defined as template SLE) toward which criteria are selected. This is a crucial step: If the template is represented by a poly-phenotypic SLE, then there is a substantial probability that the classified SLE patients enrolled into a cohort reflect the template and promote poly-phenotypic SLE variants, i.e., not compatible with a one disease entity. On the other hand, if the template SLE is a one disease entity, then the appearing classified SLE is possibly reflecting a homogeneous syndrome. This needs to be critically investigated.

This intellectual problem may, consequently, imply that there are reasons both to trust and to mistrust the validity of current SLE classification criteria (15–18, 38). Is this statistically supported philosophy productive, and does this philosophy reflect realities? We end up to ask: *How is indeed SLE as a template in the equation above defined in this important and critical classification process and is “the causality principle” implemented in this definition or not?*

The main strategy has (yet theoretically) failed because any element of the causality principle or causality cascade paradigm content have been ignored in the classification criteria selection processes. This is true because causality in a concrete and reflective context is not described in the relevant Delphi panel processes [see the central literature in (15–18, 38)]. Therefore, the clinical impact of the currently dogmatic classification criteria is theoretically uncertain and problematic.

### The abstract “template SLE” in the equation: The constructed and abstracted template SLE version reflects the basis for selection of the criteria—is this sound science or inspired consensus?

What are the authoritative instructions that dominate the classification criteria selection processes? Important premises rely in the following three questions:

- Which scientific rules related to classification criteria selection allow us to define SLE as a one disease entity in contrast to a spectrum of “SLE-like non-SLE syndromes” (2, 39–41)
- Are the criteria interrelated and interactive—in the sense of a causality cascade (13, 31, 42)?
- Is the template SLE formed by a single dominating cause (allowing us to define SLE as a one disease entity) or is SLE a poly-causal and consequently poly-phenotypic syndrome? For example, central criteria like lupus nephritis, serositis, and joint inflammation are not a consequence of a one and the same cause, but of disparate causes promoting organ-selective processes.

Therefore, the equation described above (version 1) has a parallel more complex formulation that may be more relevant for complex syndromes like SLE.

**Version 2 and an alternative interpretation:**

*A (symbolizing “criteria”) is statistically associated with B*’ *(symbolizing poly-causal and poly-phenotypic SLE) because B*’ *is the factor that promotes A.*

This implies that the SLE template is poly-phenotypic and poly-causal by nature.

**Figure 1:**
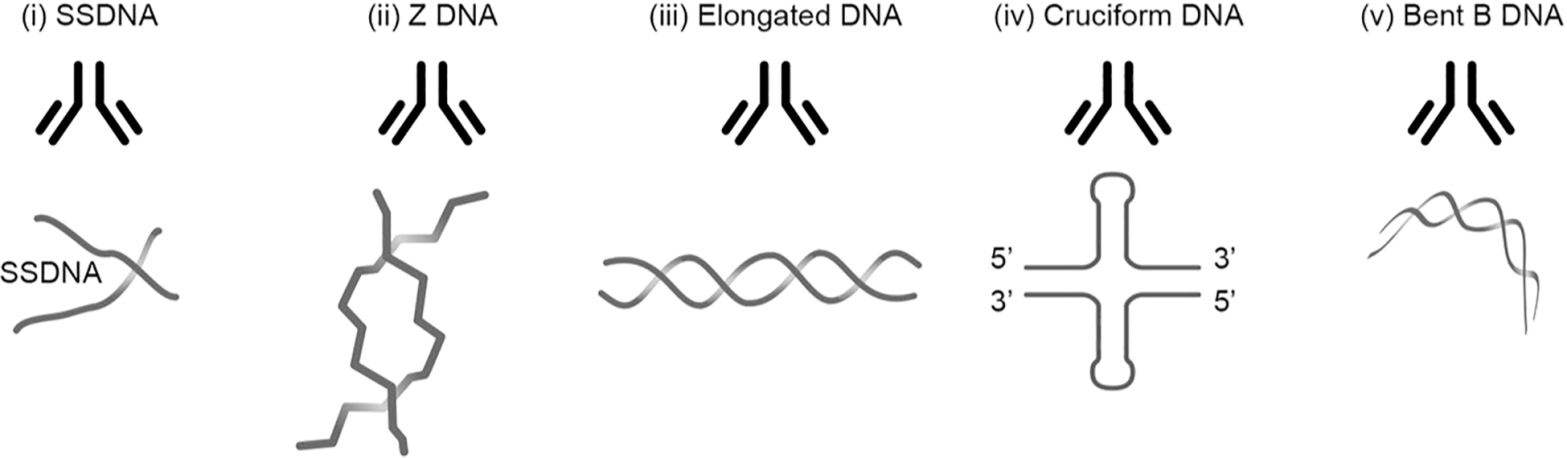
Transcriptionally active DNA expresses distinct DNA structures. Each structure is a unique antigen. (i) The B DNA helix is opened by single-strand-binding proteins (SSBP), which stabilize ssDNA. The ssDNA is involved in replication and repair. (ii) Z dsDNA is a left-handed, high-energy, double-helix DNA structure. Z DNA forms during transcription as a result of torsional strains that depend on interaction with mobile polymerases. Z DNA is associated with linker DNA. (iii) Elongated (linker) dsDNA is a relaxed and stable, right-handed, and low-energy form of B DNA. (iv) Cruciform dsDNA is another dsDNA structure and is different from B and Z DNA. Its formation requires that sequences (palindromes) in one strand are repeated on the other strand in opposite directions. The cruciform structures are, like Z DNA, higher-energy structures. **(v)** The dsDNA in core nucleosomes is defined as bent B DNA. Bent DNA is a compacted structure influenced by the histone octamer and histone H1. These structures (i–v) are unique in terms of inducing highly specific antibodies with potential pathogenic impact if chromatin fragments are exposed *in situ* (see **Table 2**). This figure demonstrates the unique immunogenic DNA structures [revised from (43)].

The two variants of the SLE template (B and B’) provoke the question whether the steadily increasing spectrum of SLE classification criteria [**Table 1**, see also **Figure 1** in (13)] reflects SLE as one disease entity or a poly-causal and poly-phenotypic syndrome denominated by the abstracted term “SLE-like non-SLE syndromes” or just “SLE-like syndromes.” This is fundamental to the question if we can define and distinguish classification versus diagnostic criteria. It all depends on whether the SLE templates B and B’—as presented in equation versions 1 and 2—reflect a single causality cascade–driven or a poly-causal driven SLE syndrome. *In conclusion, we basically need to describe and understand the nature of the SLE template implemented in criteria selection processes* (15–18, 35).

The distinctive difference between the two variants of the equation is the crucial element to understand which version(s) of SLE will be classified and made objects for research on SLE problems. The most important problem that basically confronts classification consensus deals with the *untold definition of the template SLE* used as basis for criteria selection: the B and B’ forms of SLE in equation versions 1 and 2. The lack of a well-defined clinical version of the authoritative template SLE and the marginalization or the ignorance of the causality principle (30, 32, 44) and the causality cascade paradigms (31, 42) will basically reduce usability of the operational SLE classification criteria processes responsive for delimitation of SLE. This lack of template SLE definition(s) will inevitably preserve the dogmatic character of SLE classification criteria. *SLE classification criteria are given the status as authoritative instructors to primarily classify and indirectly diagnose SLE once enrolled into an SLE cohort, without formal scientific evidence defending their authoritative status!*

### SLE classification criteria are vague and imprecise—a reflection of Delphi panel processes—strategy, competency, and relevance

Two central problems adhere to the basic procedures defining SLE classification criteria. For the first, a large panel of experts on various aspects of basic and clinical processes that constitute SLE selected and subsequently elected SLE classification criteria in harmony with standard Delphi panel procedures (36, 37). A fundamental and crucial question is aimed to clarify how implemented basic protocols define the *selection principle of SLE classification criteria (see a relevant discussion in* (36, 37). Most importantly, where can we read the details about the principle delimitations and, not least, if self-critical censorship is part of the process? The latter point is necessary if we will trust this clinically important project.

The expert (Delphi) panel is represented by scientists with insight into the complex research foci covering SLE: Genetics, clinics, basic etiology, and pathogenesis. *Each member is an individual with own ideas, opinions, and scientific interests and priorities, possibly with unique hypotheses and insight and with personal integrity^2^.*

This manifold is at the same time a threat and a challenge to develop consensus strategies and solutions. The same can be said for statistical testing of new criteria versions against the foregoing ones and against a selected SLE cohort, an ideal version of SLE with status as a template^3^ SLE syndrome. Is this template described, if not, why? At least, efforts to do so should have been attempted. The acceptance and the impact of these SLE classification criteria versions may give birth to new dogmas, relevant also for other aspects of classified SLE syndromes.

If we consider the definition of SLE as “poly-etiologic,” “poly-causal,” and “poly-phenotypic,” then it is understandable why a new dogma started to dominate the philosophical considerations: Because of the “poly-” situation, it is now consent around a new authoritative dogma. *This dogma states that it is not possible to develop diagnostic criteria*, because diagnostic criteria are based on causality (23, 30, 32, 44), which is not implemented in the Delphi panel processes. The causality principle links causality to the causality cascade (31, 45, 46), a paradigm that is empirically very similar to the causality cascade activation of the complement system (47, 48) or activation of the coagulation system (49). Theoretically, in SLE, mono- or oligo-causal events trigger specters of symptoms and parameters that are interdependent and interactive in form of downstream cause-effect-cause networks—an instructive principle to describe interactions that ends in the interactive causality cascade. From this, we can make the following distinctions.

*Classification criteria are collected without reflecting or implementing clear delimitating pathogenic intrinsically individual factors or rules:* The criteria are not selected on the basis of cause-effect paradigms and are not characterizing SLE as a one disease entity (see above, the “poly-characteristics”).

*Diagnostic criteria, on the other hand, relate to* the Koch-Pasteur Transformation paradigm: Studying causes of infections rather than their symptoms (23, 50). This means, in relation to SLE, that we have to search and describe the SLE-related causality and define the SLE diagnosis based on the causality cascade. This will reduce number of true SLE cases but increase cases labeled as “SLE-like” or “SLE-like non-SLE” syndromes.

*To implement disease initiation and disease prolongation and expansion elements, processes in monogenic SLE* (51–54) *in contrast to polygenic SLE* (55) *may be a valid prototype reflective model to study a causality-driven disease process.*

## Are SLE classification criteria met with cogent and influential skepticism: what are the arguments *pro et contra*?

Dogmas are the bases for conflicts between those who defend and those who criticize their impacts—whether they are substantiated by hypotheses or by idiosyncratic dogmatic statements. These conflicts are obviously counterfactual and counterproductive and do not serve science well. In this context, it may be wise to reinstate and restore the theories of radical science-based paradigm shifts described by TS Kuhn in 1962 (56). A central and instructive example of a science-based paradigm shift is new scientific traditions introduced by Robert Koch and Louis Pasteur. They presented significant changes in our thinking of disease etiology through the implementation of the modern version of “the causality principle (30).: Importantly, they influenced all aspects of today’s medical concepts and research strategies: The transformation of medical science from studying symptoms to study their causes [discussed and reviewed in (50, 57)]. *This is a highly relevant and practicable guide into solutions of the true impact of dogmas in all aspects of medical research.*

Although easy to acquire and rely on, the immediate prognosis for implementing this transformation paradigm into critical scientific investigations of SLE may not be good, rather pessimistic. A dogma is more and more secured in “*believe in evidence-based facts.*” Implementation of critical investigations focusing on a dogma’s basic impact and functional consequences has been procrastinated, but, if realized and activated, goes into the roots of problems that define dogmas and thereby defines why we do not understand SLE. For example, what is the systemic definition of SLE used as template for SLE classification criteria identification? Transformation of studying disease phenomenology to study disease causality may be the paradigm shift that consequently may reduce the impact of SLE classification criteria. *In this horizon, can we see the glimpse of unified cause-related SLE cohorts?* It is honestly regretful to necessitate expression of this perspective—it is, indeed, obvious according to science history and science theory.

### A consequence: what ties the spectrum of SLE classification criteria together and why have classification criteria reached a status as an authoritative dogma when there is no formal evidence that supports this status?

When we carefully study the methodology and the consequent processes for SLE classification criteria selection, a clear and logic question may be formulated: What ties the spectrum of SLE classification criteria together, and do they reflect a unified causality cascade (31, 42). Clearly linked to this problem is the missing definition of SLE as a “template SLE syndrome.” In other critical words, have we identified evidence that link each of the classification criteria to each other and directly to an abstracted template form of SLE, very much like denominating SLE as “a one disease entity”? *In sum, are all accepted SLE classification criteria interdependent and interactive*? This is required if they are inherent parts of a diagnostic and, consequently, causality cascade. However, interdependency and interactivity of criteria is not a requirement if we analyze the relevant published SLE classification publications!

What is then the SLE classification criteria defining or classifying—”SLE and SLE-like non-SLE syndromes”? The contour of SLE classification criteria as a functional dogma is more and more intrusive but also interpretatively more and more authoritative. From these considerations, *the validity and authority of SLE classification criteria stand or fall with a clear definition of SLE as template for the SLE classification criteria selection processes*. The diffuse definition of a classified SLE syndrome makes diagnostic criteria more relevant. The argument that it is not possible to develop diagnostic criteria for SLE is valid as long as SLE classification criteria identify and define the SLE cohorts, and at the same time exclude SLE as “a one causal disease entity.” This situation requires reinvestigation of central elements linked to SLE classification, SLE diagnostics, and, equally important, “the template SLE syndrome.”

It is not verified that the SLE classification criteria versions have served SLE research well—this is an assumptive statement that is not confirmed by data. This skepticism is expressed in the title of a recently published manuscript: “Why is it so difficult to understand why we do not understand SLE: Facts, conflicts, and implementation of the causality cascade paradigm” (13). What is the evidence for the statement saying that the classification criteria have served SLE research well, as long as the criteria most probably classify SLE as “poly-phenotypic” and “poly-causal”?

## SLE classification as a tentative dogmatic process—an evolution confronting a termination

The next stage of this discussion focuses on dogmatic processes, with a focus on the controversial implementation of diagnostic criteria in SLE research. Classification criteria do not have impact as diagnostic criteria, although they ultimately serve as diagnostic criteria once a lupus patient is classified and enrolled into an SLE cohort (23). This is a paradox with consequences *as long as it is not settled that the criteria are linked to each other in chains of events that are interactive, consistent with the causality cascade paradigm* (31, 42).

An initial cause promote one or few effects, each of these effects (or some of them) may have the potential to transform from being effects to act as (neo-) causes with further downstream alternating cause-effect-cause events. In this example, each of the downstream cause-effect factors is interactive and interconnected (31, 45). As is discussed above, it is surprising that causality and the causality cascade paradigms have not been problematized and considered in Delphi panel processes, as is evident when we analyze the core SLE classification criteria publications (15–18).

As described above, the today’s SLE classification criteria harbor and functionally promote inherent problems that theoretically challenge the value of contemporary scientific studies focusing on SLE cohorts. There is no formal evidence against this rational reasoning and interpretation!

SLE expert (Delphi) panels suggest criteria believed to be linked to SLE. This is a strategy that challenges scientific realities. For the first, the four central SLE classification criteria versions share many of the individual criteria [see **Table 1**, and an extended discussion in (13)]. There is no revolutionary paradigm shifts (56) or considerate symbolic or factual development among the criteria versions, but a modest linear evolution. This is signified by a statistically significant correlation between the criteria versions (15–18). Again, the core question will be: How are these SLE patients diagnosed? By classification criteria or by identification of, and correlation with, an abstracted SLE template configuration? So far, this makes the prognosis for a development of diagnostic criteria pessimistic. *Diagnostic criteria are still not visible in the horizon.*

### Are SLE classification criteria approaching the end of its impact?

Why can we classify SLE by criteria but not diagnose SLE by criteria (4)? In other terms, what is the difference between the two classes of criteria?

The easy answer is that classification criteria is heterogenic and not selected by a down-stream interactive causality cascade–related biological process (31). This means that classification criteria do not reflect “a one cause principle” but embrace different ones, indicating that the classified clinical syndrome is heterogenic (the poly-paradigm) in contrast to the “a one disease entity” paradigm [(2); discussed in (5, 23)]. This is an important concept: *Classification criteria do not reflect and identify a causality cascade–promoted SLE.*

**Figure 2:**
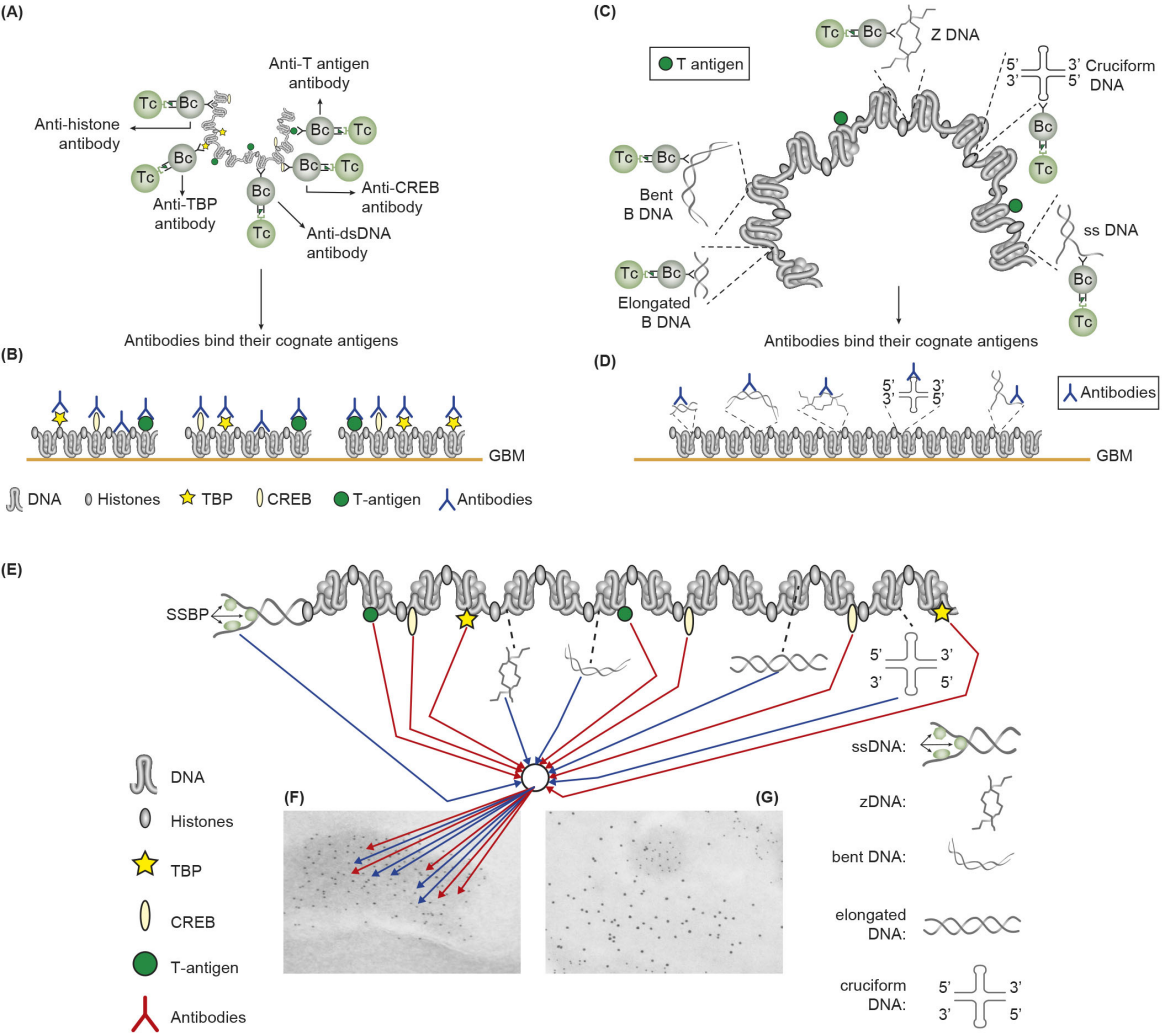
Induction of anti-chromatin antibodies **(A)** and anti-DNA structure-specific antibodies **(B)**—demonstration of Sercarz’s hapten-carrier theorem involving expression of e.g. polyomavirus T antigen as T helper cell-stimulating immunogenic carrier protein. **(A)** Injection in non-autoimmune mice with plasmids expressing polyomavirus DNA-binding T antigen induces production of antibodies to DNA, T antigen, mammalian histones, and certain transcription factors like TATA-binding protein (TBP) and cAMP-responsive element-binding protein (CREB) by cognate interaction between chromatin specific B cells and polyomavirus T antigen peptide–specific helper T cells. **(B)** These antibodies bind chromatin-antigens exposed in GBM and promote nephritis. **(C)** Identical immunization regime induces autoantibodies against elongated B DNA, bent B DNA, Z DNA, cruciform DNA, and ssDNA. **(D)** The anti-DNA structure-specific antibodies promote nephritis by binding exposed DNA antigens in GBM. Autoimmune B cells and operational immune T cells cooperate in this model. **(E)** All the induced anti-chromatin/anti-DNA structure antibodies have pathogenic potentials if binding exposed chromatin in, e.g., GBM, as is demonstrated in GBM as electron-dense structures [EDS in panels **(F, G)**]. The induced autoantibodies (stained with 5-nm gold particles) bind chromatin fragments **(F)**. Chromatin fragments are surrounded by un-affected GBM structures that bind anti-laminin antibodies added to the section *in vitro* [10-nm gold particles; **(G)**]. **(A, D)** is modified by combining **Figures 4**, **5** from (43).

From this contemplation, diagnostic SLE criteria must on the other hand reflect a unifying basic cause that triggers an interactive and interdependent causality cascade [see an example of an SLE derived, diagnostically useful causality cascade, **Figure 2**, in (23)]. This may have as a consequence that classified SLE embraces disparate SLE variants, whereas SLE diagnosed by composition of causality cascade–associated interactive criteria may result in selection of “a one disease entity”—similar condition. This may mean that *core interactive and inter-related criteria* embody stringent markers for SLE that may fulfill the definition of pathogenic-based criteria. The central contemplative question that we have to consider is therefore the following: *Are true, logic, and operational SLE classification criteria representing effects of disparate inciting causes? This is a key question that we need to answer if we will transform SLE as a dogmatic syndrome into an evidential fact-based template SLE syndrome—a syndrome where the causality principle and the causality cascade paradigm are central diagnostic-related elements that can be applied in future SLE research.*

## The dsDNA, anti-dsDNA antibodies, and lupus nephritis—a history of rise and fall of dogmas and realities

If historical data are forgotten, ignored, or abandoned, then research progression may suffer and being hampered and disoriented with respect to science-based insight. Research efforts must therefore reconstruct, reclaim, and revitalize the potentially important but ignored data.

Relevant in this context is a discussion around DNA structures; their instrumental involvement in the genetic machinery; and their immunogenicity, origin, and immuno-pathological impact. In order to review and reconsider history, the following information is important to settle.

*The anti-dsDNA antibodies were originally described in association with bacterial infections*. These historically important but ignored and forgotten observations are dated back in 1938–1939 (58–60). From a modern and updated understanding of anti-DNA assay principles (43, 61, 62), we have to admit that we do not know the specificity and nature of these infection-associated anti-DNA antibodies [see basic information in (43)]. It is, however, a remarkable experience to comprehend that, over time, these first anti-DNA antibodies did not remain as a focus for scientists. They were rarely cited in the relevant literature over decades to follow.

Later, however, bacterial DNA entered an important scientific scenery as a central structure with potential to induce anti-dsDNA autoantibodies similar to those encountered in SLE—the bacterial DNA structure (63, 64). The excellent and eye-opening studies of Pisetsky and coworkers have demonstrated that bacterial DNA structures have the potential to induce true autoimmunity to DNA, including mammalian dsDNA (65–68), with a clear link to SLE-related immunopathology.

The silence around the 1938–1939 observations was not terminated in 1957 when anti-dsDNA antibodies were for the first time described in sera of SLE patients. After the 1957 publications, we heard very little from the1938–1939 observations, while the focus on the 1957 publications soon reached status as legendary dogmas: Anti-DNA antibodies became authoritative markers for SLE. These were, fascinatingly enough, described simultaneously by four independent research groups (69–72). With those discoveries, anti-dsDNA antibodies were for the first time connected with a clinical condition, although no one anticipated the enormous impact of these discoveries for future research. This development is tightly associated with Paul Ehrlich’s “horror autotoxicus” paradigm [a neologism introduced by Paul Ehrlich that means “the horror of self-toxicity,” discussed in (73, 74)]. The overall evolutionary history of DNA and chromatin autoimmunity has been reviewed in several comprehensive studies (7, 43, 75–79) and will not be further summarized or discussed here.

The discovery of the archetypical DNA structure (80–83) and subsequent anti-DNA immunity and autoimmunity (a distinction that link DNA to conventional immunity and to autoimmunity) started a new scientific era that revolutionized the molecular bases of inheritance [reviewed in (7, 77)] but also fertilized a strong and vivid development of new, persistent, and distracting dogmas.

These dogmas have had negative effects on research efforts aimed to understand the nature of SLE. Above is presented a comprehensive discussion aimed to understand negative consequences of prevailing dogmas in SLE, with a dominant focus on SLE classification criteria and how they inherit principles that inevitably lead to scientific confusions. These dogmas embrace several authoritative statements that have hampered SLE research—so also functional insight into DNA autoimmunity.

In the following, authoritative dogmas linked to definition of DNA structure, immunogenicity of DNA, diagnostic impact of anti-DNA antibodies, and their pathogenic effects in kidneys will be discussed.

## Definition of DNA

Regarding the SLE classification criteria, it is strange to read that anti-dsDNA antibodies are selected as an SLE criterion without limitations. Until mid-1990s, anti-dsDNA antibodies were regarded unique to SLE, causing nephritis, but with poorly defined mechanism(s); and dsDNA was immunologically inert. What is the definition of dsDNA today? In the context, dsDNA is a counterproductive term that uncovers lack of insight that ignores central historical and contemporary data on DNA structures. Already Franklin and Goslin described in 1953 existence of two DNA forms, the A and B forms (80). Subsequently, a variety of distinct DNA structures have been described and published (84): Elongated (linker) DNA (85, 86), bent DNA (87, 88), Z DNA (89–91), single-stranded DNA (ssDNA) (92–94), cruciform DNA (95–97), and other dynamically changing DNA structures linked to their function in the genetic machinery [**Table 2**, **Figure 1**, discussed and reviewed in (43)].

**Table 2:** DNA structures formed *in vivo* are immunogenic but are not fully investigated for clinical specificities.

DNA structure	Immunogenic?	Ref	Autoimmunogenic?	Clinical specificity	REF	Comments	Ref
B DNA elongated	Yes#	(98–100)	Yes	SLE* Infections, cancer	(4, 7)	Inconsistently investigated	(43)
B DNA Bent	Yes	(98, 101)	Yes	SLE* Others?	(102, 103)	Inconsistently investigated	(43)
ssDNA	Yes	(104, 105)	Yes	Clinically unspecific	(43)	Even detected in healthy individuals	-
Cruciform DNA	Yes	(106)	Not determined	Not examined	N/A	Unknown clinical specificity	(43)
Z DNA	Yes	(107, 108)	Yes	SLE-other	(109, 110)	Needs more clinical studies	(43)
Viral DNA**	Yes	(111–113)	Not actual per definition	Autoimmunity in viral infections	(99, 101, 111, 114–116)	May induce e.g. anti-dsDNA antibodies by hapten-carrier mechanism*	(111, 114, 117)
Bacterial DNA	Yes	(63, 64)	Not actual per definition	Autoimmunity in bacterial inf.	(58–60, 63, 64, 68, 118, 119)	May induce autoantibodies	(43, 63, 120)

*Depends on control groups. **Refers to polyomavirus DNA. ^#^Elongated B DNA is immunogenic provided it is in complex with an immunogenic carrier protein in context of the Sercarz’s hapten-carrier theorem (121, 122). This is valid for all the DNA structures listed.

In order to unequivocally improve insight into the theoretical and de facto impact of such DNA structures and corresponding antibodies in clinical medicine, time has now come to scientifically focus on this potentially wide and important research field (43). Because all the described DNA structures are immunogenic (**Table 2**), we need to develop assay systems that implement analytical conditions that expose each of the individually central DNA structures. Such assay systems have been discussed recently (43).

Why is this analytical field up till today ignored in clinical medicine and rheumatology? One obvious explanation is that the SLE classification versions published in 1982 (16), 2012 (17), and 2019 (18) presented anti-DNA antibodies as the slogan-like “*the anti-dsDNA antibody.”* This has the error-prone consequence that the anti-dsDNA antibodies are detected by “dsDNA-specific analytical assays.” This is inconsistent; each individual analytical principle may allow detection of unique DNA specificities. For example, anti-elongated dsDNA antibodies are tested by Enzyme linked immunosorbent assay (ELISA), whereas the Farr assay in high salt may detect anti-Z DNA and not necessarily high avidity anti-B DNA antibodies as claimed before (79, 102). For details on these analytical principles, see a discussion presented in (43). Thus, different contemporary assay systems relate to the dogmatic terminology—”the anti-dsDNA antibody”—without further distinctions. This is a clear example that a dogma has misled scientists to analyze avidity and impact of “the anti-dsDNA antibody” rather than to split this term into “concise anti-DNA sub-specificities” with disparate immunogenic potentials, and each with inherent but various clinical impacts (**Table 2**).

In conclusion, we need to update, rethink and reinvestigate impact of anti-DNA structure-specific antibodies to reveal the erroneously authoritative impact of the dogmatic version of *“The anti-dsDNA antibody”*!

### Clinical specificity of anti-dsDNA antibodies—dogmas and challenges

As the term “dsDNA” was counterproductive in immunology and immunopathology, the term dsDNA/DNA was among the most important in research fields like genetics, biochemistry, and inheritance. The irrelevance of the term “dsDNA” in clinical immunology has its equivalence in the term “the clinical specificity of the anti-dsDNA antibody”—they embody similar counterproductive problems. There are few published clinical studies on impact of anti-DNA structure-specific antibodies in rheumatologic diseases/syndromes.

Mostly, the diagnostic impact of anti-DNA antibodies are described under the idiom “diagnostic impact of the *anti-dsDNA antibody*.” With some few exceptions (109, 110), we have poor insight into the diagnostic impact of antibodies against individual DNA structures [see data in **Table 2**, and also a highly relevant discussion by Rojo et al. (123, 124)]. Again, “The dsDNA” paradigm forms also here a dogma that hampers structural and critical research on the clinical impact of anti-DNA antibodies (43). What we know today is that “the anti-dsDNA antibody” is linked to SLE, infections and malignancies (7). Whether this relates also to antibodies against disparate DNA structures is poorly investigated and mostly unknown.

### Nephritogenic anti-dsDNA antibodies

There are two mainstream hypotheses that link anti-dsDNA antibodies to the lupus nephritis pathogenesis. Both these hypotheses share the status as dogmatic: They have been proposed over decades but have, however, never been subjected to rigorous comparative analyses to determine if one or the other—or both models—are correct (77).

#### The anti-chromatin antibody model

The chromatin structure with all its accessible surface exposed ligands may be recognized by specific autoantibodies that recirculate in SLE patients. From this, two conclusions may influence mode of lupus nephritis incitement and progression. The first is that all ligands toward which antibodies can be specific for must have been accessible for ligand-specific B cell antigen receptors, like DNA sequences or DNA backbones, histones and non-histone regulatory enzymes, and proteins. The second conclusion is that the induced antibodies may access the same ligands on chromatin fragments exposed in glomerulus basement membranes (GBMs) and in the mesangial matrix. This may mean that many different anti-DNA/anti-chromatin antibodies have nephritic potentials (detailed in **Figure 2**). The extensive repertoire of nephritogenic anti-chromatin antibodies embodies anti-DNA structure-specific and anti-chromatin ligand antibodies (**Figure 2**). This model inherit elements of a dogma, as concise molecular aspects of lupus nephritis promoted by anti-dsDNA antibodies and other chromatin antibodies have not been sufficiently investigated (77, 125–128). It is, for the following discussion, important to define anti-chromatin antibodies, in general, as principally nephritogenic, not only, as regularly stated, anti-dsDNA antibodies.

#### Cross-reactive antibodies complicate interpretation of the anti-chromatin antibody model

There is no consensus as to describe how anti-dsDNA antibodies promote lupus nephritis (77). Still there exist two interpretative hypothesis-based directions. One is the anti-chromatin antibody model [see above (126, 129)]. An alternative model has been announced and discussed over decades (77, 126–128, 130), implying that a cross-reactive pattern of anti-dsDNA antibodies has gradually created a new paradigm (see references in **Table 3**). This implies that anti-dsDNA antibodies cross-react with inherent regular structures in GBM and in the mesangial matrix, like laminin, entactin, and collagen [ (132, 135, 145, 148–150); discussed in (77, 151, 152)]. The dogmatic character of these two models has precipitated controversies that have not released investigations aimed to conclusively verify which—or both—of the models are correct. The problems are not resolved and are currently largely ignored! Today, there is no visible research program in the horizon that may solve the problem of whether and how cross-reacting anti-dsDNA antibodies are involved in lupus nephritis. Dual specificity of nephritogenic antibodies for dsDNA and non-dsDNA membrane constituents is intriguing but does not by itself solve any aspect of this problem. Cross-reaction does not inform which of the alternative antigens are targeted *in vivo*!

**Table 3:** Examples of anti-dsDNA antibodies that cross-react with non-DNA structures.

Anti-dsDNA antibody cross-react with	References
α-Actinin	(131)
α-Actinin	(132)
Laminin	(133)
C1q	(134)
Several cross-reactive activities presented at “Fifth International Workshop on anti-DNA anti- bodies in London 2002 to highlight relevant properties of pathogenic anti-DNA antibodies”	(135)
Laminin	(136)
Nucleosomes	(137)
Platelet integrin GPIIIa49-66	(138)
TLR 4	(139)
NR2 glutamate receptor	(140)
Cell surface proteins	(141)
Ribosomal P protein	(142)
Collagen IV	(127)
Pneumococcal antigen	(143)
EBNA	(144)
Entactin	(145)
Entactin*	(146)
Phospholipids	(147)

*Mono-specific anti-entactin antibody is included to suggest a control non–cross-reactive antibody to determine if it needs dsDNA as a cross-reactive specificity to gain pathogenic potential.

#### A comparative analysis of “the chromatin” and the “cross-reacting” models

How do the chromatin and the cross-reacting models comply with the clinical course of lupus nephritis as a two-phased disease form? The following characteristics of the two models may be of interpretative help.

**Figure 3:**
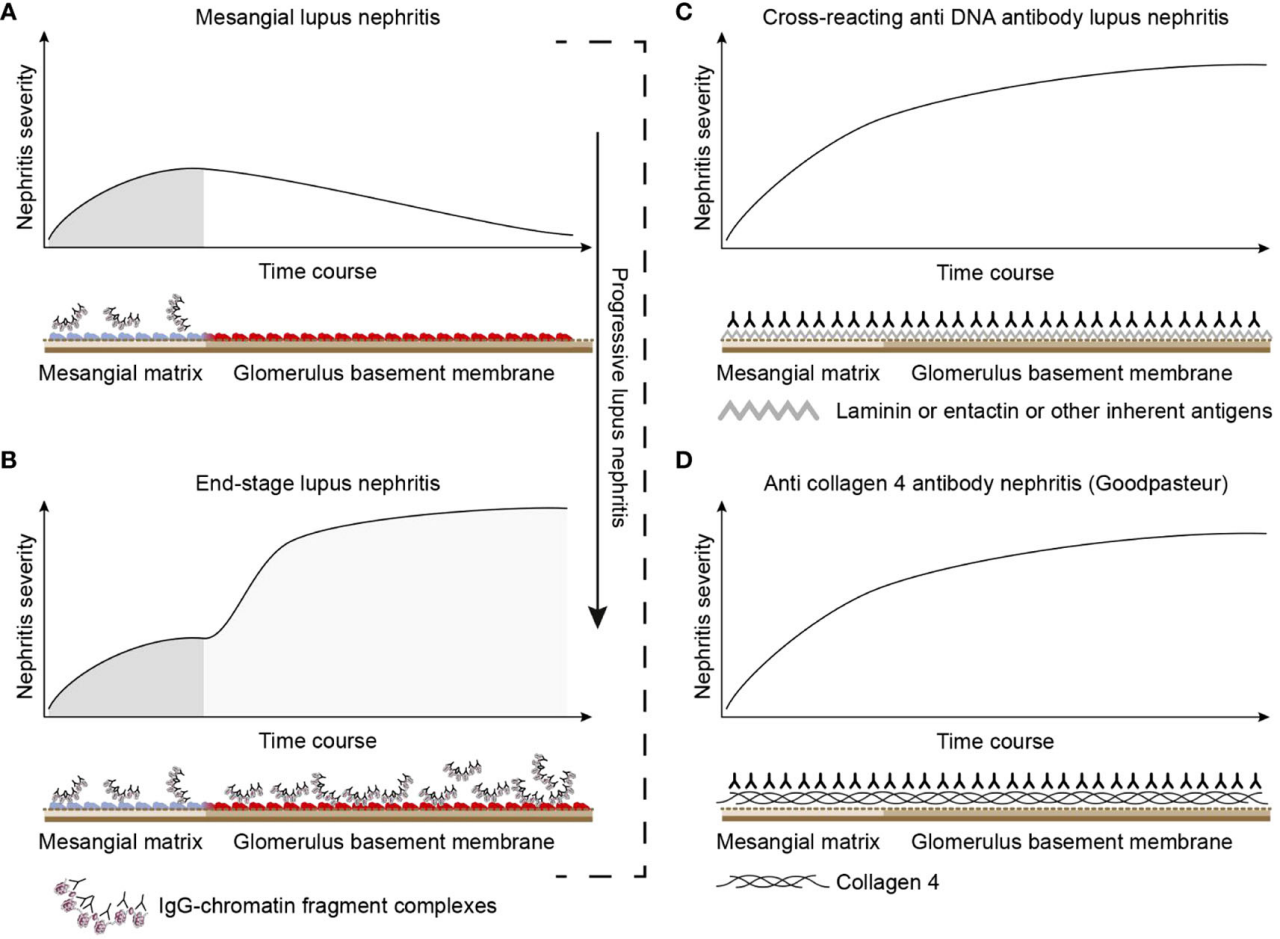
Theoretical disease profiles differ depending on the molecular specificities of the autoantibodies. **(A)** Anti-dsDNA antibodies form immune complexes with assumed circulating small chromatin fragments that accumulate in glomerulus mesangium (153). This promotes mild, early mono-phasic, and transient lupus nephritis (phase 1). Under certain conditions, mesangial inflammation promotes silencing of the renal *DNase 1* endonuclease (153). In reflection of loss of *DNase 1* enzyme activity, large chromatin fragments released from dead cells accumulate as undigested large chromatin fragments in complex with anti-chromatin antibodies in mesangial matrix and in GBM [see details in (153)]. This promotes end-stage nephritis (**B**; phase 2)—*a second-phased progression of lupus nephritis.* This biphasic lupus nephritis model contrasts the cross-reacting model (described in panel **C**). Here, a cross-reacting anti-DNA antibody binds inherent membrane antigens (like laminin, collagen, or entactin) shared between the mesangial matrix and GBM. Therefore, the nephritis profile is mono-phasic, as the mesangium and GBM are simultaneously affected by antibodies. This mono-phasic nephritis is complementary to nephritis in Goodpasture syndrome **(D)**. Goodpasture-type nephritis is caused by anti-collagen IV antibodies that bind collagen structures shared by the mesangial matrix and the GBM. The antibodies therefore promote a mono-phasic nephritis profile as in the cross-reacting lupus nephritis model similar to the Goodpasture syndrome. This figure was copied from (6).

**Figure 4:**
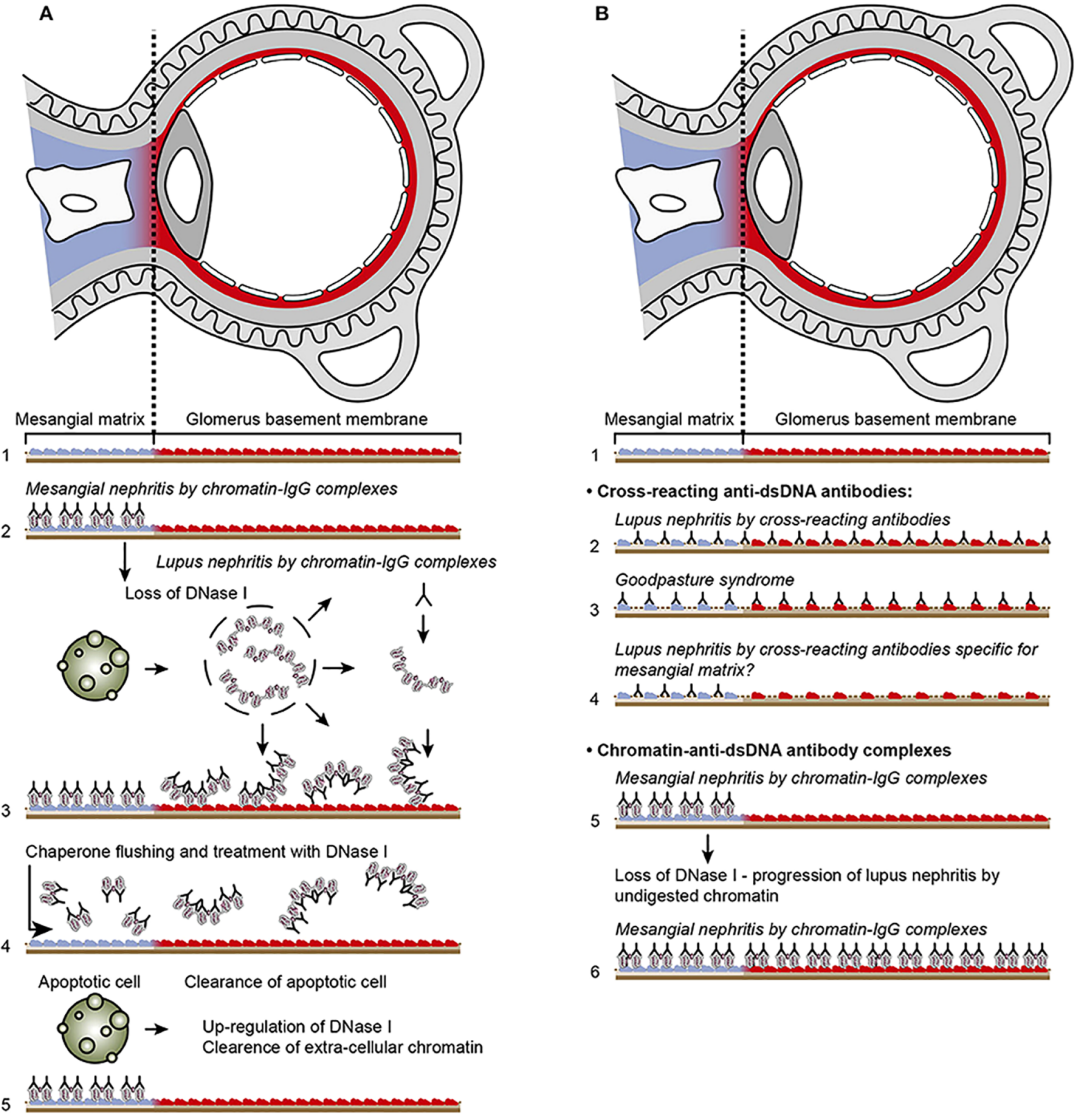
Principal problems to be solved before the chromatin or the cross-reactive model for lupus nephritis can be settled. In **(A)**, the chromatin model is presented. On top, a principal presentation of the architecture of a glomerulus is described. In line 1, the mesangial matrix (blue) and its transition into the GBM (red) is principally demonstrated. In a classical progression of lupus nephritis (153, 154), chromatin-IgG complexes deposit in the mesangial matrix and form the early mesangial nephritis (line 2). One consequence of this limited inflammation is silencing of the renal endonuclease *DNase 1*, a consequent reduced fragmentation of chromatin from dead cells, and a subsequent deposition of large chromatin fragments in complex with IgG within the GBM [line 3 (153, 155)]. This forms the process that promote glomerular inflammation and progression of lupus nephritis into end stage disease [discussed in (77)]. Silencing of *DNase 1* expression in this situation is unique to the kidney and does not occur in other organs (155). Because chromatin-IgG complexes bind laminins and collagens in the GBM with relatively high affinity (156) and are released locally in the glomerulus, these observations may explain the canonical progression of lupus nephritis as described by Weening et al. (154). This process may have therapeutic consequences, because chromatin prone to be deposited in GBM may be removed by flushing kidneys with the negatively charged heparin or other analogous chaperone molecules [line 4 (157)], and, theoretically, the process may be interrupted upon upregulation of renal *DNase 1* expression [line 5 (157)]. In **(B)**, the glomerulus architecture is organized as in **(A)**, and the matrix-GBM transition is principally illustrated (line 1). In the cross-reacting model, cross-reacting anti-dsDNA antibodies bind intrinsic glomerular structures like entactin, laminin or collagen (line 2). Because these antibodies may bind ligands shared by mesangial matrix and GBM, the antibodies are expected to bind simultaneously in the mesangial matrix and in the GBM (line 2). Therefore, the cross-reactive antibodies might well-initiate a glomerular inflammation more similar to the renal inflammation in Goodpasture syndrome (line 3) than to the stepwise progression of lupus nephritis as illustrated for the chromatin model illustrated in **(A)**, lines 2 and 3. This difference illustrated in panels **(A, B)** has not been considered in the relevant literature. One possible exception for this Goodpasture-like inflammation would be an early production of antibodies specific for a ligand unique for the matrix (suggested in line 4). In contrast to this hypothetical cross-reacting model, lines 5 and 6 summarize progressive lupus nephritis according to the chromatin model. These principally conflicting models are summarized in **(A)**, lines 2 and 3 for the chromatin model, and in **(B)**, line 2 for the cross-reactive model. This figure is a revised and extended version of **Figure 4** in the work of Rekvig et al. (158) with permission from Elsevier (license number 4832930988362).

The chromatin lupus nephritis model is described as a progressive profile that implements two immunopathological phases: phase 1 and phase 2 (**Figures 3A, B**; see mechanistic details in **Figure 4A**). Phase 1 is caused by an early and low production of anti-dsDNA/anti-chromatin antibodies. The sole presence of the anti-dsDNA antibody promotes accumulation of immune complexes in the mesangial matrix—the immune complexes consisting of the antibody and recirculating low levels of chromatin fragments [for details, see (153)]. This process confers to clinically silent or mild mesangial nephritis with low-graded proteinuria [**Figure 3A** (154)].

**Figure 5:**
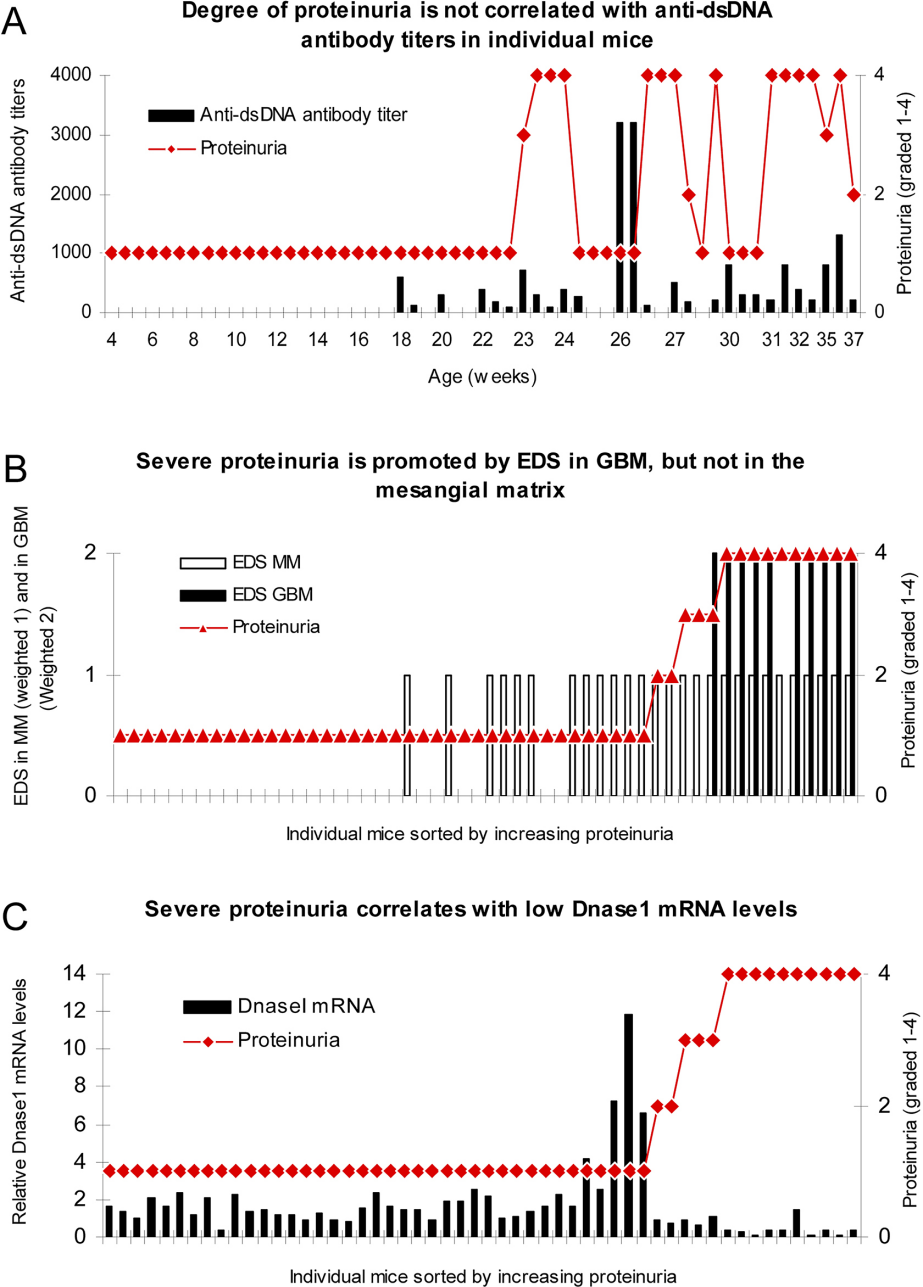
Severe proteinuria correlates with EDS deposits in GBM and inversely with renal DNase1 mRNA levels. In mice sorted for age, there was no association between degree of proteinuria and levels of anti-dsDNA antibody titers **(A)**. To analyze if location of electron dense structure (EDS, i.e., chromatin fragments) deposits had impact on proteinuria, data on proteinuria and deposition of EDS in the mesangial matrix (weighted 1 in **B**) or in the GBM (weighted 2 to make a visual distinction from deposits in the mesangial matrix, **B**) were combined for each mouse and sorted by ascending values of proteinuria. Severe proteinuria (≥20 g/L) was, except for one mouse with intermediate proteinuria (≤3 g/L), exclusively observed in mice with EDS in GBM **(B)**, whereas intermediate or mild proteinuria was observed in only 4 out of the 17 mice with mesangial matrix deposits **(B)**. In panel **(C)**, degree of proteinuria and renal DNase1 mRNA levels were paired and sorted by ascending proteinuria. Severe proteinuria (≥20 g/L) correlated with a substantial loss of DNase1 mRNA (and enzyme activity). Thus, in mice selected for proteinuria ≥20 g/L, renal DNase1 mRNA was nearby lost in all but one mouse **(C)**, and deposits of chromatin-IgG complexes (observed as EDS) in GBM were observed only in these mice. This figure is copied from PLOS One; https://doi.org/10.1371/journal.pone.0008474.g004.

The immune complex deposition in the mesangial matrix (phase 1) have been shown to promote a consequent local inflammation that simultaneously confers to an abrupt silencing of the renal *DNase 1* gene (illustrated in **Figures 5A–C**), the dominant renal endonuclease) (155, 159). This confers to increased glomerular accumulation of large (less digested) chromatin fragments in complex with immunoglobulin G (IgG) anti-dsDNA antibodies in the GBM (phase 2, **Figure 3B**; details in **Figures 5A–C**). This pattern complies with progression of lupus nephritis into end-stage organ disease (153). *Thus, the chromatin model implies development of a biphasic progressive lupus nephritis model (as illustrated in* **Figures 3B** *)*.

**Figure 6:**
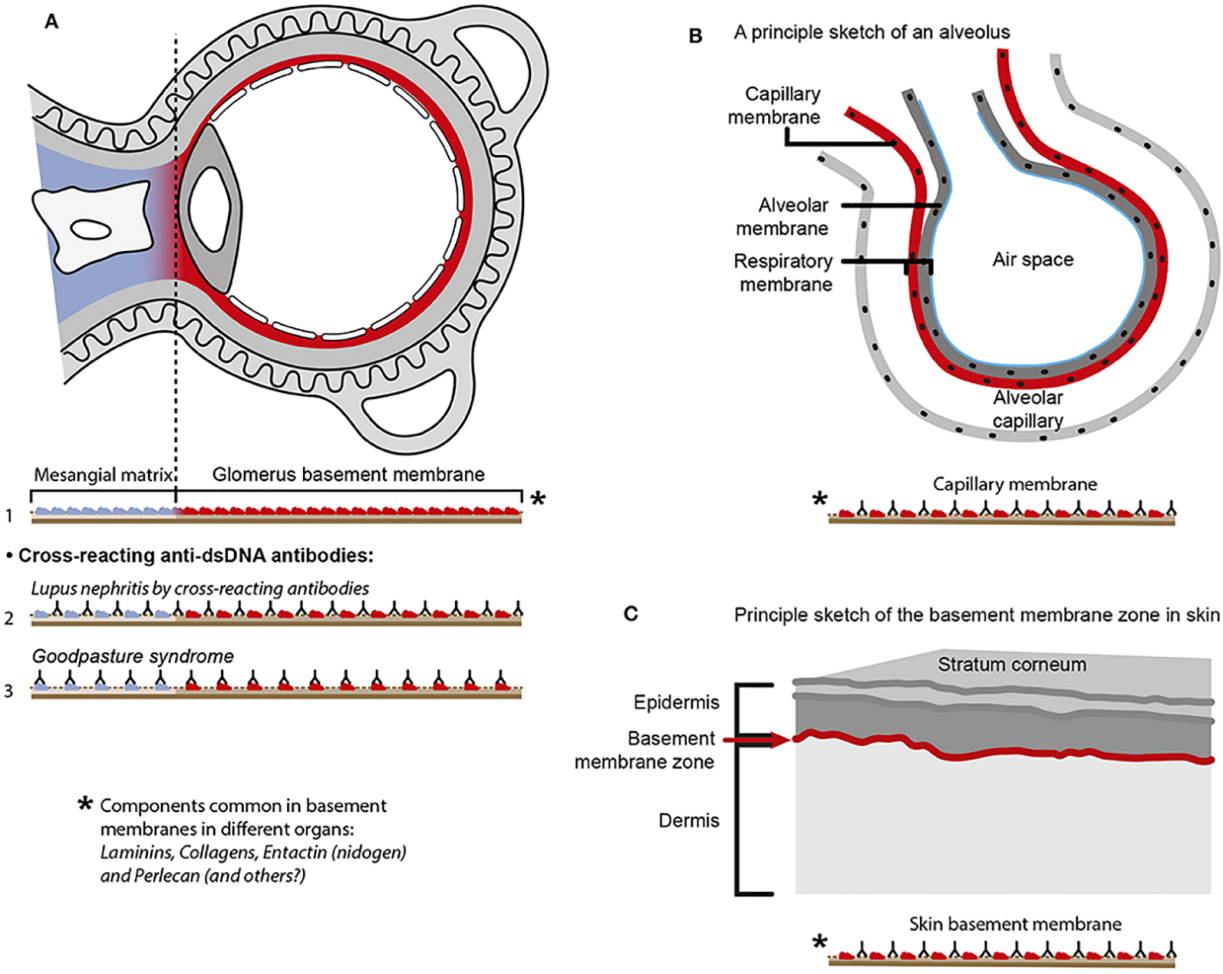
Principal problems linked to the cross-reactive model for lupus nephritis. The cross-reactive model inherits a provoking problem. Laminins, entactin, and collagens are obligate constituents in all basement membranes. This is relevant for basement membranes in glomeruli [see (77) for discussion], alveoli (163), and skin (164). Accordingly, one should expect affection of glomeruli **(A)**, alveoli **(B)**, and skin **(C)** in analogy to Goodpasture syndrome [glomeruli and alveoli (165, 166)] and autoimmune skin diseases (167, 168). Surprisingly, in the context of studies on the impact of cross-reactive anti-dsDNA antibodies as a model for pathogenesis of lupus nephritis, the involvement in other organs has not been discussed or considered in the relevant studies. Observational and experimental studies argue against this theoretical model. Analyses of nephritic glomeruli by electron microscopy (EM), immune EM (IEM), co-localization IEM, and TUNEL co-localization IEM allowed clear indications that *in vivo* bound IgG were observed in electron dense structures (EDS) localized in the matrix and GBM (169–171), as summarized and discussed in (77). These EDS were TUNEL-positive, and bound antibodies against histones, transcription factors, and dsDNA were added to sections *in vitro*. This figure is a reprint with permission from Elsevier (license number 4832930988362)].

The cross-reacting lupus nephritis model presents a quite different theoretical dynamic nephritis profile (**Figures 3C**, **4B**). This is explained by an equal distribution of cross-reacting target membrane ligands like laminin, collagen, and entactin in the mesangial matrix and in the GBM [reviewed in (160–162)]. The target for cross-reactive molecules is shared by GBM and the mesangial matrix on one side [**Figure 6A** illustrates cross-reactive anti-dsDNA antibodies in the mesangial matrix and in GBM (172)]; **Figure 6B** illustrates that cross-reactive antibodies bind the same antigens shared between GBM and the alveolar or shared between GBM and skin membranes (**Figure 6C**) on the other side (167, 173). An interpretation of these data will ultimately end as a concept where the nephritis profiles are shared between the cross-reacting lupus nephritis model and the autoimmune Goodpasture syndrome [indicated and compared in **Figure 3C**, the cross-reacting model, and **Figure 3D**, Goodpasture nephritis (see the mechanistic cross-reactive model illustrated in **Figure 4B**)]. The two latter pathogenic situations are principally similar, as the target antigens in both situations are shared between the glomerular mesangial matrix and GBM (174, 175). This model confers to the cross-reacting nephritis model as a one-phased dynamic nephritis, in contrast to the factual evolution of nephritis as described in (NZBxNZW)F1 mice, the two-phased chromatin model (153).

From these considerations, the following considerations may be emphasized. Three patterns may be informative in order to develop sound research hypotheses to identify how anti-dsDNA antibodies exert their nephritogenic activity:

- The dynamic and progressive nature of lupus nephritis complies with the chromatin model (see details in **Figures 3A, B**) and not with the cross-reactive model (**Figures 3C, D**); see also comparative details illustrated in **Figure 4A** versus **4B**.
- Structure and composition of immune deposits in glomeruli are informative. These structures have been shown to constitute chromatin fragments in the mesangial matrix and in the GBM, to which anti-dsDNA antibodies and other anti-chromatin antibodies co-localize (153, 169, 176). It is highly unlikely that all these anti-chromatin antibodies cross-react with membrane antigens. The chromatin fragments are accumulating in membranes and matrices due to the fact that the renal *DNase 1* endonuclease gene and enzyme activity in kidneys is suppressed in the course of early lupus nephritis [**Figures 4**, **5**; discussed above and in (77)]. This endonuclease deficiency correlates with severely progressive lupus nephritis [ (153, 177); discussed in (77)].
- The following data argue against the cross-reactive model: If anti-dsDNA antibodies cross-react with, e.g., entactin (145) or laminin (133, 178–180) and if such antibodies access entactin and laminin *in vivo*, then why are not these antibodies also binding laminin and entactin in, e.g., alveoli of the lung and in the skin [**Figures 6**; membrane characteristics and composition in this structures, see (146, 160, 161, 167, 172, 173)]? In other words, if anti-dsDNA antibodies cross-react with membrane ligands shared between lungs and kidneys, then they can hypothetically be anticipated to share features imposed by Goodpasture antibodies against collagen IV (165, 174). There are no such studies or observations reported.

### Conclusions on DNA, anti-dsDNA antibodies, and lupus nephritis: I am right! am I? what is the evidence?

A conclusion on problems linked to dsDNA, anti-dsDNA antibodies, and lupus nephritis requires that we identify authoritative dogmas that lack identifiable evidence for their approval. We strongly need to generate strict science-based hypotheses that are not formulated to serve the purpose: *I am right*, but to describe real evidence-based natural processes at basic molecular levels. There are many intellectually based conflicts in the aftermath of studies described above.

There is, after all these years, imperative to study (and solve) these dogma-related problems in detail. We need to describe the real nature of pathogenic processes, the hypotheses, and doubts, and conflicts, as described in this analysis. We have to accept that SLE is significantly affected by unproven dogmas, but SLE is also like a fertile landscape for growing critical hypothesis-based scientific activities. Much research needs to be performed in times to come!

### SLE-related dogmas and paradoxes—their existence are imperative arguments to develop critical hypotheses

A practical and vital consequence in science theory is that *scientific hypotheses that are not objects for critical investigations are in danger of being transformed into empirical dogmas*. These theoretical dictums are relevant when we consider our lack of understanding of SLE. Still, hypotheses can be justified if we implement better definitions that consider relevant causal knowledge (30). From this reasoning, dogmas come, as a consequence of new (immature) discoveries, and go, if the discoveries are proven *either real or wrong* by clear and relevant evidence. In the latter two *real or wrong* situations, dogmas transform into proven realities and leave their status and definition as dogmas. Linked to this, the more a dogma is defended despite lack of convincing evidence, the stronger will its dogmatic character manifest itself. This is why SLE classification criteria is given a dogmatic status in this study.

## A final emotional dilemma

For how long time shall we declare that SLE is an enigmatic syndrome, with an enigmatic origin, with an unpredictable progression, and with a poor diagnostic approach. Considering the continuous refinements of the SLE classification criteria over the last 50 years, the unbearable weight of optimism expresses that SLE will sooner or later be defined, delimitated, and understood. When reading the current relevant literature, this optimism is realistic if we can collaborate within the system science paradigm^4^. Maybe we even today have sufficient insight and concrete knowledge to promote fertile insight and erudition to promote insightful paradigm shifts, but this activity requires courage and endurance and a “testable faith” in own ideas.

## Concluding remarks

In the present study, dogmas that affect our understanding of what SLE is have been discussed, and some central ones were identified. Many of these dogmas relate to identification and selection of classification (and putative diagnostic) criteria. A central problem describes if these criteria are related to the causal principle (mimicking symptoms) in SLE. Assumedly, the criteria may by themselves account for pathophysiological processes, but not necessarily in an interactive perspective. The next central question is if the criteria reflect one or disseminated cause-promoting syndromes. This is a central question that may categorically separate SLE into “a one disease entity” or into a poorly defined “template (archetypical) SLE,” “SLE-like,” or “SLE-like non-SLE” syndromes. It is in the context important to determine if SLE is a one cause driven syndrome or if SLE represents disseminated poly-causal syndromes. This has not yet been thoroughly discussed, investigated or published in the relevant literature. This may fulfill SLE classification criteria as an unproven dogma.

The distinction between “template SLE,” “SLE-like,” or “SLE-like non-SLE” syndromes may be of substantial importance if we aim to generate homogeneous SLE cohorts ideal for penetrating studies of new experimental therapy modalities, causality, genetics, pathophysiology and diagnostics.

***Concrete conflicts discussed here:***

- The first conflict is that SLE classification criteria are selected to be central symbols for SLE, as they are promoted and selected by expert panels, although stringent causality rules have not been implemented. Therefore, these symbols have a status closer to be dogmas than to reflect evidence-based facts. The criteria do not inherit strong unifying causal impact in any direction. SLE-related dogmas are not delimitated by the causality principle. No evidence has been provided that the dogmas described here are really proving if factual and evidence-based effects or processes describe the real nature of SLE.
- The second aspect is that each SLE classification criteria version is statistically correlated with former versions of criteria. **Table 1** describes, however, that criteria are reiterated in the progressive versions over 5 decades (1971–2019). This helps to explain the significant correlation between the criteria versions. SLE, as a poorly defined syndrome (<i>SLE is an enigmatic prototype autoimmune syndrome), defines patients to be enrolled into SLE cohorts; however, an SLE template model has not been clearly and understandably defined in such relevant studies.
- The third aspect is that we need to unequivocally define “the template SLE” that may be central for classification criteria selection. In the present study, the following equation is formulated [modified from (13)]:

*A (symbolizing “criteria”) is statistically associated with B’ (symbolizing poly-causal and poly-phenotypic SLE) because B’ is the factor that promotes A.*

This equation demonstrates that the selected classification criteria and “the template SLE syndrome” are influencing each other as two-sided mirror images. This scenery, defined by the equation referred to above, is not influenced by the theoretically important causality principle (46).

An intellectual approach is to rethink and reinterpret central information, in order to develop a new radical version of SLE defined as “an identifiable and delimited template SLE model.” In this model, the causality principle and the processual down-stream cause-effect-cause paradigms in terms of a causality cascade may be identifiable as a rational bases for critically investigating dogmas, and to transform them into rational causality-dependent insight. A dominant focus will be to select operational and logic causal SLE classification criteria.

The ultimate conclusion of this study is that we still have a long way to go to prepare bases for new critical and productive hypotheses and radical new and productive research models in order to comprehend what SLE really is.

### Central taking home messages

- In centrum of SLE problems: Despite intense research over decades, SLE is still described as an enigmatic autoimmune syndrome. This is linked to how SLE is classified and diagnosed. Critical hypotheses have until today not been formulated to solve this problem.
- The scientific impact of SLE classification criteria has not been critically discussed. They are randomly selected, as the causality principle has never been introduced as a central element. This leaves an open question whether these criteria are interrelated and interactive as in a causality cascade.
- Anti-dsDNA antibodies are not specific for one DNA specificity—the mammalian B DNA. They are specific for several disparate specificities (ssDNA, elongated dsDNA, Z dsDNA, Cruciform dsDNA, and Bent dsDNA). These are not investigated in a diagnostic or a pathogenic context
- Anti-dsDNA antibodies are (still) claimed to be specific for SLE. It has, however, since 1938–1939 been known that these antibodies are produced in infections and for decades also in malignancies to report central links
- In the end, a central decisive question to contemplate: Why are SLE diagnostic criteria disregarded in SLE research? This question needs an explanation and a conclusion!

## Data Availability

The original contributions presented in the study are included in the article/supplementary material. Further inquiries can be directed to the corresponding author.

## Footnotes

1 Originally published in (13)

2 This may be problematic to consider if we reflect on the often-experienced contradictive referee statements, revealing that manuscripts are rejected by one referee, whereas accepted by another! This reflects contrasting intuition, knowledge or priorities.

3 Template is a term that critically reflects or states *“a molecule (as of DNA) that serves as a pattern for the generation of another macromolecule (such as messenger RNA), or something that establishes or serves as a pattern”* (Merriam-Webster).

4 System science is an interdisciplinary field that studies the complexity of systems in nature, social, or any other scientific field (Columbia University Mailman School of Public Health).
